# Pleiotropic Roles of *uvrY* on Biofilm Formation, Motility and Virulence in Uropathogenic *Escherichia coli* CFT073

**DOI:** 10.1371/journal.pone.0055492

**Published:** 2013-02-01

**Authors:** Arindam Mitra, Senthilkumar Palaniyandi, Christopher D. Herren, Xiaoping Zhu, Suman Mukhopadhyay

**Affiliations:** 1 Virginia-Maryland Regional College of Veterinary Medicine, University of Maryland, College Park, Maryland, United States of America; 2 Maryland Pathogen Research Institute, University of Maryland, College Park, Maryland, United States of America; Loyola University Medical Center, United States of America

## Abstract

Urinary tract infections primarily caused by uropathogenic strains of *Escherichia coli (E. coli)* remain a significant public health problem in both developed and developing countries. An important virulence determinant in uropathogenesis is biofilm formation which requires expression of fimbriae, flagella, and other surface components such as lipopolysaccharides. In this study, we explored the regulation of *uvrY* and *csrA* genes in biofilm formation, motility and virulence determinants in uropathogenic *E. coli*. We found that mutation in *uvrY* suppressed biofilm formation on abiotic surfaces such as polyvinyl chloride, polystyrene and glass, and complementation of *uvrY* in the mutant restored the biofilm phenotype. We further evaluated the role of *uvrY* gene in expression of type 1 fimbriae, an important adhesin that facilitates adhesion to various abiotic surfaces. We found that phase variation of type 1 fimbriae between fimbriated and afimbriated mode was modulated by *uvrY* at its transcriptional level. Deletion mutant of *uvrY* lowered expression of fimbrial recombinase genes, such as *fimB*, *fimE*, and *fimA*, a gene encoding major fimbrial subunit. Furthermore, transcription of virulence specific genes such as *papA*, *hlyB* and *galU* was also reduced in the deletion mutant. Swarming motility and expression of *flhD* and *flhC* was also diminished in the mutant. Taken together, our findings unravel a possible mechanism in which *uvrY* facilitates biofilm formation, persistence and virulence of uropathogenic *E. coli*.

## Introduction

Uropathogenic *Escherichia coli* (UPEC) causes urinary tract infections (cystitis, pyelonephritis), and septicemia in humans and animals [1], [2]. Zoonotic transmission of antimicrobial-resistant strains of UPEC from animals to humans was proposed [3]. UPEC adherence to urogenital epithelial cells is the first step in the initiation of infection, which is generally accomplished by type 1 fimbriae [4], [5]. In UPEC, the initial process of attachment is mediated by several adhesins, of which, type 1 and pap fimbriae play a critical role in colonization in urinary bladder and kidneys respectively. Type 1 fimbriae mediate UPEC attachment to the bladder epithelium by binding to mannose-containing glycoproteins and promote the early stages of biofilm formation on both biotic and abiotic surfaces [6]. Furthermore, the fimbriae exhibit phase variation by inversion of a 314-bp DNA element (*fim* switch), which harbors the promoter for several structural proteins [7], [8]. This allows (“on” orientation) or prevents (“off” orientation) transcription of the structural genes in *E. coli*.

Bacterial biofilms are structured bacterial communities embedded in a self-produced exopolysaccharide matrix on biotic and abiotic surfaces [9], which are, indeed, the major problem of the interference of bacterial eradication with antibiotics. An important hallmark of UPEC is the formation of biofilm, which facilitates UPEC strains to persist in the urogenital tract and interfere with bacterial eradication [10]. Biofilm formation in *E. coli* requires a set of gene expressions facilitating its initiation, attachment and subsequent maturation. For example, a variety of virulence factors in *E. coli*, such as hemolysin, fimbriae, lipopolysaccharide (LPS), secreted proteins, capsules and iron-acquisition systems [11], [12], which allow bacterial colonization in the mucosal epithelial cells lining the urogenital tract, invade, and further form intracellular biofilm-like pods in uroepithelial cells [13]. Remarkably, UPEC is capable of forming biofilms on the abiotic surfaces of indwelling medical devices such as catheters [14], [15], which lead to “Catheter-Associated Urinary Tract Infection” (CAUTI) in clinics [16]. CAUTI is especially fatal in immunocompromised, debilitated and diabetic patients [17], [18]. Therefore, biofilm formation in UPEC is important for causing persistent colonization in bladder, kidneys or urine and in hospital settings.

The two-component regulatory systems (TCSs) are a ubiquitous mechanism for coupling various environmental stimuli with the transcription program of bacteria [19], [20]. A typical TCS consists of a sensory protein kinase and a cognate response regulator [19]. The sensory protein kinase monitors environmental signals and transduces the information, via a phosphorelay system, to the response regulator, which, in turn, responds and modulates gene expression. The BarA-UvrY TCS in *E. coli* is pleiotropic and have been linked with several metabolic processes including biofilm formation, oxidative stress, sigma S expression, and efficient adaptation in carbon utilization [21], [22]. In this system, BarA is the sensor kinase and UvrY is the cognate response regulator [23]. In addition, a critical downstream effect of the BarA-UvrY TCS in *E. coli* is its regulation on Carbon Storage Regulatory system (Csr) [22]. Csr has been shown to control the ‘switching’ of *E. coli* from a colonization state to a persistent state [24]. The Csr system in *E. coli* is composed of the 61 amino acid CsrA protein and two small, non-coding regulatory RNAs, CsrB and CsrC [25]. Transcription of these two small RNAs is regulated by the BarA/UvrY TCS system in *E. coli*. In this circuit, UvrY enhances transcriptions of CsrB and CsrC which in turn bind and interfere with the activity of RNA binding function of the CsrA. CsrA also regulates the TCS and subsequently controls its own expression in an auto-regulatory loop. It has been shown CsrB and CsrC RNA's contain several degenerative sequences that serve as multiple binding sites for CsrA protein. In addition, CsrA can directly target several *E. coli* genes, such as the *glg* operon which encodes genes in glycogen biosynthesis and the *pgaA* mRNA which encodes a polysaccharide adhesin involved in biofilm formation [26]–[28].

In this study, we investigated the roles of *uvrY* and *csrA* in regulating expression of genes relevant to *E. coli* biofilm formation. Our previous study has shown that mutants deficient in *barA* and *uvrY* genes of avian pathogenic *E. coli* are attenuated in an avian embryo model, exhibited reduced persistence in liver, spleen and decreased invasiveness in chicken embryo cells (12). This attenuation is correlated with the down regulation of fimbriae, exopolysaccharide accumulation and sensitivity to oxidative stress *in vivo* [29]. Our recent findings further verify that mutation in either *barA* or *uvrY* of UPEC CFT073 results in reduced persistence of mutants in bladder, kidneys or urine in an ascending murine UTI model [30]. Because UPEC share similar genes with APEC [31], [32], both studies lead us to hypothesize that the BarA/UvrY/Csr pathway plays an important role in the biofilm formation of UPEC. To prove this hypothesis in UPEC, the goal of this study is to determine the relationships between the BarA/UvrY/Csr pathway and regulation of genes relevant to *in vitro* biofilm formation of the CFT073 strain. Since fimbriae is a common adhesin present in both commensal and pathogenic *E. coli* [5], we also compared the regulation of type 1 fimbriae by the BarA/UvrY/Csr system between *E. coli* and UPEC CFT073 strains. To our knowledge the relationship of the BarA/UvrY/Csr system with the fimbriae in regulating the biofilm formation has not been previously explored. Our results indicated that the BarA/UvrY/Csr system could play key roles in uropathogenesis of UPEC in regulating the expressions of genes relevant to the biofilm formation at both transcriptional and post-transcriptional levels.

## Materials and Methods

### Bacterial strains, plasmids and oligonucleotides

Bacterial strains and oligonucleotides used in this study are listed in Table 1 and Table 2, respectively. Sequenced strains of uropathogenic *E. coli* CFT073 and non-pathogenic *E. coli* K-12, MG1655 were chosen for genetic manipulation in this study [33], [34]. Precise in-frame deletions were constructed in *E. coli* K-12 and UPEC CFT073 by λ Red recombinase method [35] as previously described [30]. PCR verification and phenotype characterization were performed to confirm the deletions. TR1-5 MG1655 was a kind gift of T. Romeo [36].

**Table 1:** Bacterial strains and plasmids used in this study.

Strains	Genotype	Source
CFT073	Wild-type Uropathogenic *E. coli*	H. L. Mobley [33]
SM3010	*ΔuvrY*::*cam*	CFT073
SM3011	*ΔcsrA*::*cam*	CFT073
SM3013	Δ*uvrY*::*cam*/p-uvrY	SM3010
SM3014	Δ*csrA*::*cam*/p-csrA	SM3011
MG1655	Wild-type K-12 λ- rph-1	D. J. Jin [34]
AM1002	Δ*uvrY*::*cam*	MG1655
AM1005	Δ*uvrY*::*cam*/p-uvrY	AM1002
TR1-5MG1655	Δ*csrA*::*kan*	T. Romeo [36]
AM1006	Δ*csrA*::*kan*/p-csrA	TR1-5 MG1655
**Plasmids**		
pBR322	Cloning Vector	Invitrogen
pSM2	pBR322 containing uvrY gene; Ap^r^	[29]
pSM7	pBR322 containing csrA gene; Ap^r^	[29]
pBB2-1	*fimA-lacZYA* on pPR274	William R. Schwan [40]
pWS124-17	*fimA-lacZYA* locked on on pPP2-6	William R. Schwan [40]

**Table 2:** List of oligonucleotides used in the study.

Primer designation	Sequence (5′-3′)	Gene/target sequence
OSM64	CCCGAATTCATAATTTCATCGTAGGGCTTACTGTGA	*uvrY*
OSM65	CCCCTGCAGATGCACGCCTGGCTGGGTTAC	
OSM250	AGCGTTCTGTAAGCCTGTGAAGGT	*rrnA*
OSM251	TAACGTTGGACAGGAACCCTTGGT	
OSM260	ACCGTTCAGTTAGGACAGGTTCGT	*fimA*
OSM261	TCTGCAGAGCCAGAACGTTGGTAT	
OSM271	GGAATCGGTGTAGATGTAACCCC	*Icd*
OSM272	CGTCCTGACCATAAACCTGTGTGG	
OSM287	GCTCGTCACGGTCGCAACAA	*lrhA*
OSM288	ACATCCAGCGCTAATTTCGG	
OSM295	CAGTAATGCTGCTCGTTTTGCCG	*fim* promoter [39]
OSM296	GACAGAGCCGACAGAACAACG	
OSM297	CGACAGCAGAGCTGGTCGCTC	*fim* switch orientation [39]
OSM298	GTAAATTATTTCTCTTGTAAAT	
	TAATTTCACATCACCTCCGC	
OSM299	GCGGAGGTGATGTGAAATTAA	
	TTTACAATAGAAATAATTTAC	
OSM309	ACTCTGCGGACCACTTGGGA	*papA*
OSM310	CCAACTATTCCTCAGGGGCA	
OSM315	GATGAAACCGCAGAAGGCTT	*kpsE*
OSM316	GCGATGCGATGTGACATCTC	
OSM347	CAAGGGCGCTGGTGAACAAC	*hlyB*
OSM348	AACAGGAACTCGCTGAACCC	
OSM351	AGTTCGTCAGGGTCTGGCGA	*galU*
OSM352	CAACGCCATATGCGGTCACA	
OSM357	CCATGATGCAGGCGGTTTGT	*fimE*
OSM358	CCACGGCTTCACGCTCATCA	
OSM359	GCCAAAGCAAAACCACACGA	*fimB*
OSM360	AACGCACCCGCTATTGAACA	
OSM363	TTTCATGGTCTGCGTGTTAGTG	*ipuA*
OSM364	TTACCCGCAGCAGAAACTATGT	

The *uvrY* gene was amplified in the divergent *yec*F promoter using primer pairs OSM 64 and OSM 65 (Table 2). The amplified product was cloned into vector pCR2.1 using TOPO-TA cloning method (Invitrogen, Carlsbad, CA). Plasmids were sequenced to confirm the amplification and cloning. A 700-bp *BamH* 1-*EcoR* V DNA fragment was cloned into pBR322; the open reading frame (ORF) of the *uvrY* gene was placed in the same orientation as the *tet* gene in the vector.

### Assessment of biofilm formation by crystal violet staining

Biofilm formation was evaluated on three abiotic surfaces - PVC, Polystyrene, and borosilicate cover glass as previously described [37]. Strains were grown in Luria broth (LB) supplemented with appropriate antibiotics without shaking and diluted (1∶100) in 50 ml LB broth with necessary antibiotic and grown at 37°C for 1 hour. Approximately equal amount of cells were taken as starting culture as determined by cell density and colony forming units (CFU). To evaluate biofilm formation on abiotic surfaces such as polystyrene or PVC, microtiter plates were used. For evaluation of biofilm on the glass surface, sterile borosilicate cover slips were added in petri plates (BD, Falcon, USA). Cells were added either to sterile petri plates or to wells of polystyrene or PVC microtiter plates. The plates were incubated at room temperature for the duration of the experiment. Media was periodically removed every 24 hours by washing with 20 ml of 1× phosphate buffer saline (PBS) (pH 7.4) and fresh media was added with appropriate antibiotics. Crystal Violet staining was performed from cells grown either in polystyrene or PVC microtiter plates after forty-eight hours incubation at room temperature. Cover slips or wells were thoroughly washed with 1× PBS. After washing, cover slips were dry-fixed for 1 hour at 60°C. 0.1% Crystal Violet (SIGMA Chemicals, MO, USA) dissolved in a mixture (1∶1∶18) of isopropanol: ethyl alcohol: PBS (pH 7.4) was added either to the wells or to the cover slips and allowed to stand at room temperature for 10 min. Excess crystal violet was then completely removed by washing at least twice with PBS. The coverslips were allowed to dry, broken with a glass cutter and taken into 1.5 ml microfuge tubes. To dissolve the crystal violet dye, 33% acetic acid was added and OD_570_ value was measured with appropriate dilution. Three replicates were used per strain and experiments were repeated three times.

### Assays for *fim* switch orientation

Assays for determining the orientation of *fim* switch were performed as described earlier [38], [39]. In brief, after isolation of chromosomal DNA, 1 µg genomic DNA was used as a template to determine the “ON” and “OFF” phase respectively by using two sets of primer pairs, respectively (Table 2). In another assay, the *fimA* promoter element encompassing invertible region was amplified by PCR method. Equal amount of *fimA* promoter DNA was digested by *SnaB*1 and resolved in 2% agarose gel. The “ON” and “OFF” populations were determined by different sizes of DNA fragment either in PCR amplification or digestion experiment. These experiments were repeated three times.

### β-galactosidase assay

The *fim-lacZYA* constructs were kind gifts from Dr. William R. Schwann [40]. The *fim-lacZYA* constructs were cloned in single copy plasmid to mimic chromosomal regulation. The plasmids pBB2-1 and pWS124-17 were used to determine the relative level of *fimA* expression in various mutants and their complements. β-galactosidase assay was performed in accordance with Miller's method [41]. This assay was repeated three times with three replicates per sample.

### cDNA synthesis, quantitative RT-PCR, and real time RT-PCR

Quantitative polymerase chain reaction (qPCR) and quantitative real-time polymerase chain reaction (qRT-PCR) were performed according to the manufacturers' instructions. Total RNA was isolated in accordance with RNeasy mini protocol (Qiagen, CA) and eluted in a final 50 µl H_2_O. In brief, four ml of culture was added to 0.9 ml ice cold stop solution (Phenol∶Ethanol - 1∶19) and centrifuged at 11,000 rpm for 15 min at 4°C. Lyzozyme was used at a final concentration of 1 mg/ml to disrupt the cells. The integrity of RNA was examined by measuring OD_260/280_ ratio and by denaturing gel electrophoresis. To remove possible DNA contamination, total RNA was further subjected to a rigorous DNase (Turbo DNA-*free*, Ambion) treatment. For qPCR, the first-strand cDNA was synthesized in a mixture including 5 µg total RNA, Moloney Murine Leukemia Virus reverse transcriptase, Superscript II RnaseH^−^ (Invitrogen, Carlsbad, CA), and 50 ng random hexamers (Invitrogen, Carlsbad, CA) following the manufacturer's instructions. The quality of cDNA synthesis was determined by electrophoresis on 1.2% agarose gels and the concentration was measured by using a Nanodrop ND-1000 spectrophotometer (Nanodrop Technologies, Wilmington, DE). A 16S ribosomal RNA (*rrnA*) was used as an internal control. PCR amplification was performed for up to 30 cycles in a 25 µl total volume with Taq polymerase in a Biometra T-Gradient PCR instrument (Biometra, Horsham, PA). At various cycle intervals, a gene-specific and a control reaction tube was removed respectively. Five µl of the reaction products was resolved on a 1.2% agarose gel and the double-stranded DNA (dsDNA) product intensities were quantified using a Bio-Rad Gel Documentation system (Bio-Rad, Hercules, CA). The linear range of amplification for the *rrnA* gene was from 5 to 15 cycles in all backgrounds. The above set of samples was subjected to the qRT-PCR amplification under the identical condition in a Light Cycler (Roche, Indianapolis, IN) with SYBR Green I PCR Master Mix. The fluorescence signal from SYBR Green intercalation was monitored to quantify dsDNA product formed after each PCR cycle in comparison with a housekeeping gene, *rrnA or icd* as appropriate. This experiment was repeated three times.

### RNA stability assay

Cells were harvested for RNA isolation from late log phase during which CsrA is maximally expressed [42]. Rifampicin (Sigma, Aldrich) was added into the culture medium at a final concentration of 500 µg/ml to inhibit transcription initiation. Samples were harvested just before and after 2.5, 5, 7.5, 10 min after addition of rifampicin. The cells were harvested at 14,000 rpm in a microcentrifuge and frozen in solid CO_2_-ethanol, within 2 min. Total RNA isolation, cDNA synthesis, and qRT-PCR experiment were performed as described above. The amount of remaining *fimA* and *lrhA* mRNA was calculated from the band intensities by normalizing with intensities of housekeeping gene, *icd*. This experiment was repeated twice.

### Swarming assay

Strains were grown 48 hours under static conditions in LB broth with relevant antibiotics for three passages. Swarming assays were performed on LB media containing 0.6% Agar and 0.5% glucose. The cell number was assessed by measuring OD_600_ and by plating. To initiate swarming, 1 µl of normalized cells were stabbed into the middle of the soft agar plates and the plates were incubated overnight at 37°C. The swarming zone was photographed with an Olympus C765 Ultra zoom camera. This experiment was performed in triplicates.

### Image and statistical analysis

DNA quantification from agarose gels was performed by using the software, ImageJ. For *fim* switch orientation assay, band intensities of various strains were compared with respect to the intensity of the wild-type ON or OFF band, set at 100%. For qRT-PCR, band intensities of strains were compared to that of the wild-type set at one, normalized with a housekeeping gene, *rrnA*. All statistical analyses were performed by Graphpad Prism. One way analysis of variance followed by Dunnett's test was carried to assess statistical significance. A *p* value less than 0.05 was considered significant.

## Results

### Loss of *uvrY* abolished biofilm production in UPEC

**Figure 1:**
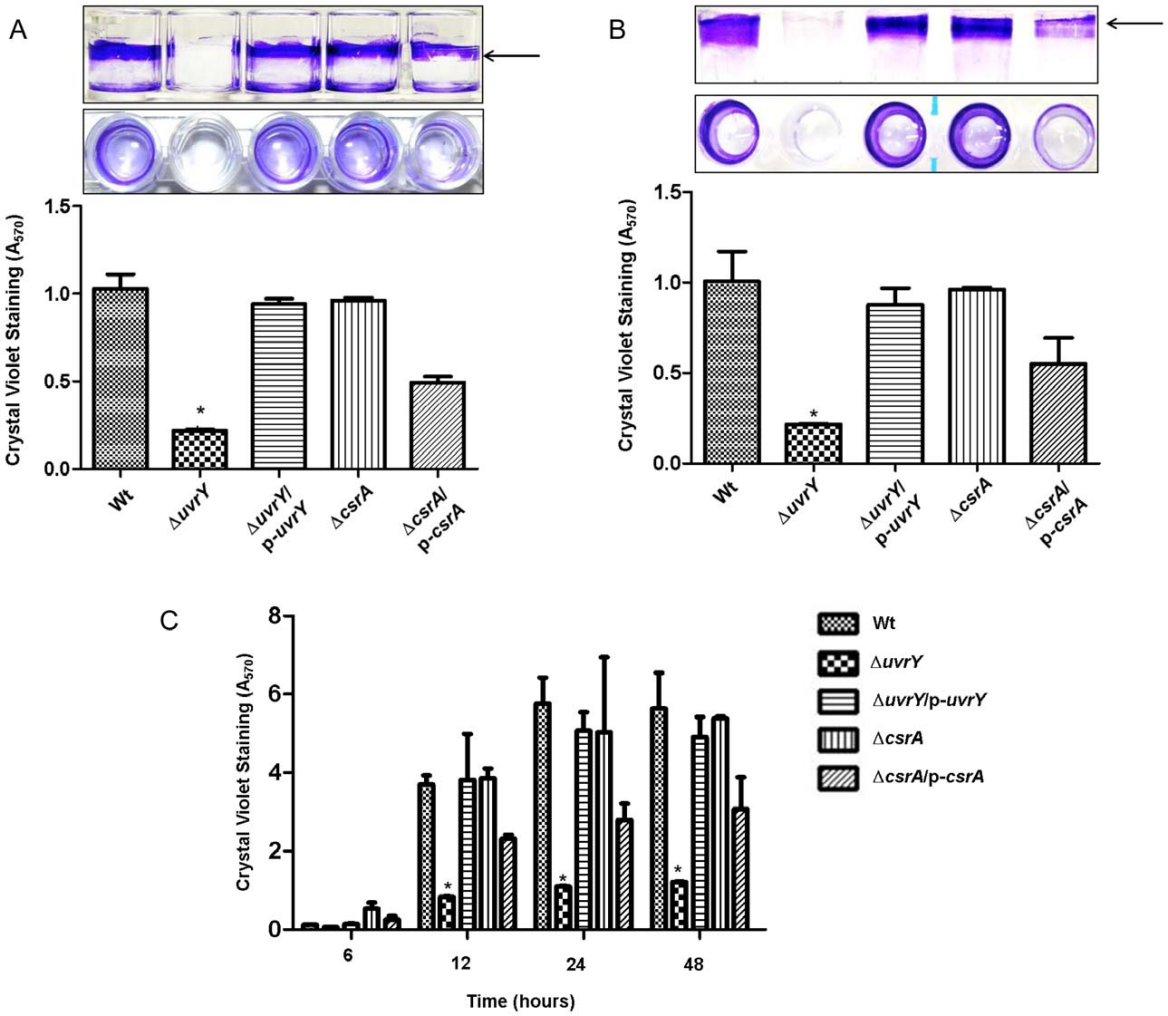
Biofilm formation on various abiotic surfaces in UPEC CFT073. Crystal violet staining was performed to assess air-liquid biofilm formation on abiotic surfaces such as polystyrene (A) or PVC (B) at 48 hours, whereas crystal violet staining was performed to assess biofilm biomass on glass (C) at 6, 12, 24 and 48 hours. Strains were grown in LB broth with appropriate antibiotics without aeration at room temperature. Bars represent means of three experiments with three replicates per sample. The error bars represent standard errors of three replicates.

To demonstrate the role of *uvrY* or *csrA* in biofilm formation, we deleted the *uvrY* or *csrA* gene in UPEC CFT073. To restore the gene function, a complementation experiment was carried out by transforming a plasmid expressing individual gene product. Our results showed that deletion of *uvrY* in CFT073 abolished biofilm production on abiotic surface such as polystyrene, PVC or borosilicate glass (Fig. 1A, B & C, lane 2), as compared to that of the wild-type (Fig. 1A, B & C, lane 1). Complementation of *ΔuvrY* in trans restored the biofilm formation to a similar level as that of the wild-type (Fig. 1A, B & C, lane 3). However, the *ΔcsrA* mutant in CFT073 showed similar levels of the biofilm production as complemented *ΔuvrY* strain (Fig. 1A, B & C, lane 4). Furthermore, complementation of *ΔcsrA* reduced biofilm production but not as low as *ΔuvrY* mutant (Fig. 1A, B & C, lane 5). Loss of *uvrY* diminished attachment to glass at all-time tested on the glass surface (Fig. 1C, lane 2). These results suggest that the *uvrY* gene product significantly facilitates the biofilm development, whereas the over-expression of *csrA* leads to inhibition of biofilm formation in CFT073 strain.

### Regulation of *fimbrial* switch by *uvrY* and *csrA* in UPEC and *E. coli* K-12

**Figure 2:**
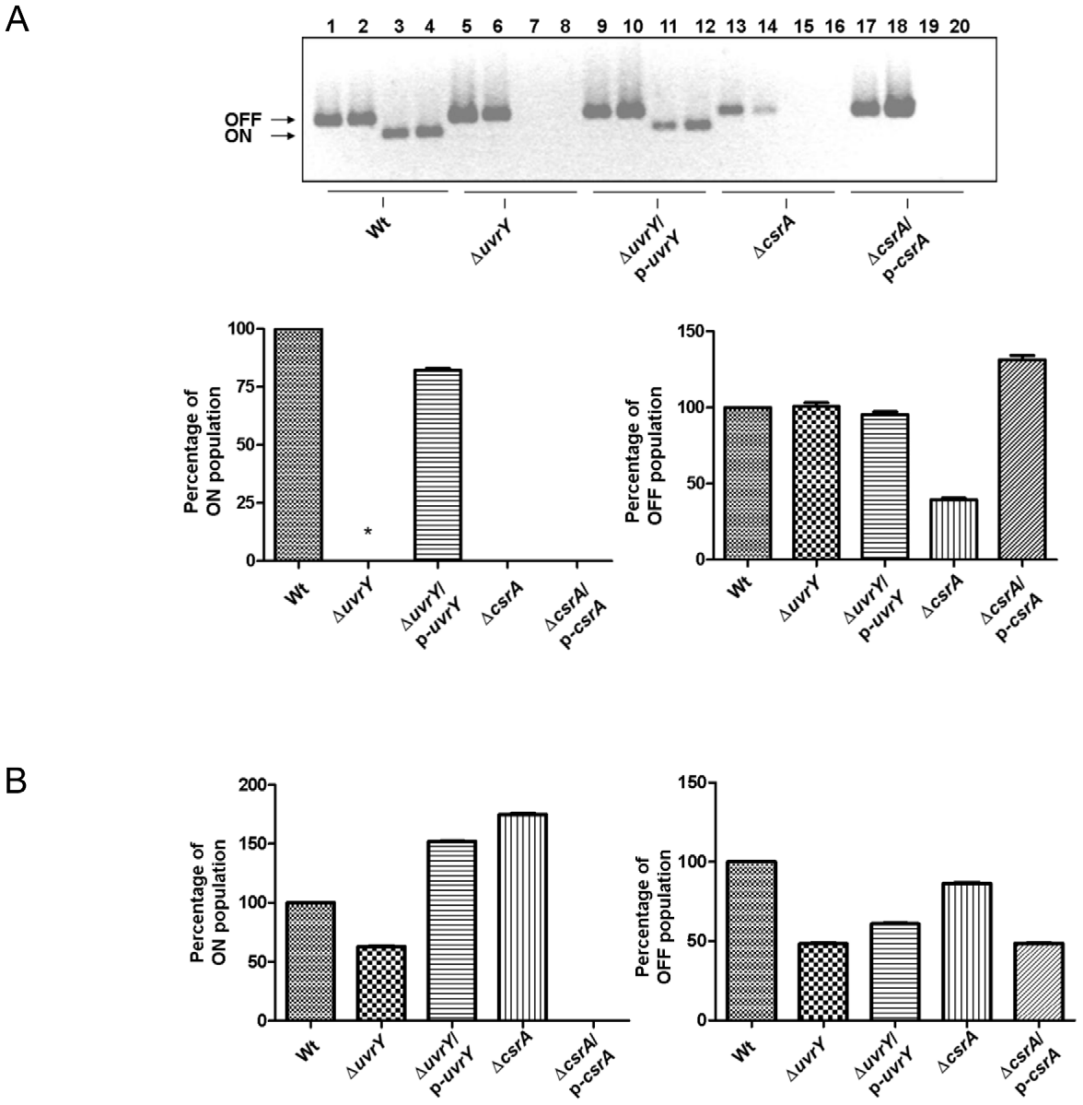
Orientation of *fim* switch in CFT073 (A) and MG1655 (B). Direction of the *fim* switch was determined by using a PCR inversion assay. Densitometric analysis was performed to assess relative intensity of bands from agarose gels by using a software, ImageJ. The OFF or ON band intensity of the wild-type was set at 100%. Two independent replicates for each strain were used for this assay. This assay was repeated three times.

An important attribute of type 1 fimbrial expression is its ability to switch between “ON” and “OFF” phase characterized by fimbriated and afimbriated phase respectively. The switch between phase OFF or ON is mediated by a 314-bp invertible element called *fimS*, which includes the *fimA* promoter flanked by 9 bp inverted repeats [43]. Site-specific recombination event alters the orientation of the promoter leading to switch between phase-ON and phase- OFF phase, thus controlling transcription of type 1 fimbriae. Two recombinase, *fimB* and *fimE*, and other regulators are involved in the regulation of this genetic switch [44]. FimB mediates interconversion between ON and OFF phase, however, FimE preferentially switches from ON to OFF phase [45]. In addition, environmental signals and DNA topology also play a role in switching the gene orientation [46]. Our data showed that mutation in *uvrY* reduced the fimbrial population (Fig. 2A & 2B, left panel, lane 2) in UPEC and *E. coli* K-12. The ON population in *uvrY* mutant was much lower in UPEC than in K-12. The function of *uvrY* was further confirmed by complementation whereby the expression of *uvrY* restored the ON population in both K-12 and CFT073 (Fig. 2A & 2B, left panel, lane 3). Mutation in *csrA* diminished the ON population in UPEC (Fig. 2A, left panel, lane 4) whereas in K-12, it enhanced the ON population (Fig. 2B, left panel, lane 4). We found that *csrA* mutant grew slower and exhibited mucoid and smooth colony phenotype. In UPEC, the overexpression of *csrA* by a plasmid expressing *csrA* gene did not restore the ON population but in K-12 the ON population was reduced (Fig. 2A & 2B, lane 5). In addition, we used single copy plasmids as well as multi-copy plasmids in our complementation experiments. Most importantly, the mutant strains were sequenced to verify the secondary mutations were around the intended sequences. However, our approach was unable to reveal whether there are mutations elsewhere in the chromosome. Anyway, these results suggest a distinct type 1 fimbrial gene regulation exists between K-12 and UPEC. Loss of *uvrY* caused a reduction in the OFF population in K-12 but not in UPEC (Fig. 2A & 2B, right panel, lane 2). Complementation of *uvrY* did not significantly alter OFF population neither in K-12 or CFT073 (Fig. 2A & B, right panel, lane 3). In UPEC, mutation in *csrA* lowered OFF population while complementation enhanced the OFF population (Fig. 2A, right panel, lanes 4&5) , the effect in K-12 was inverse (Fig. 2A & 2B, right panel, lanes 4&5). Overall, *uvrY* and *csrA* affects phase variation of fimbrial switch “ON” or “OFF” although their underlying mechanisms might be different.

### Mutation in *uvrY* influences transcription of adhesins in UPEC or *E. coli* K-12

**Figure 3:**
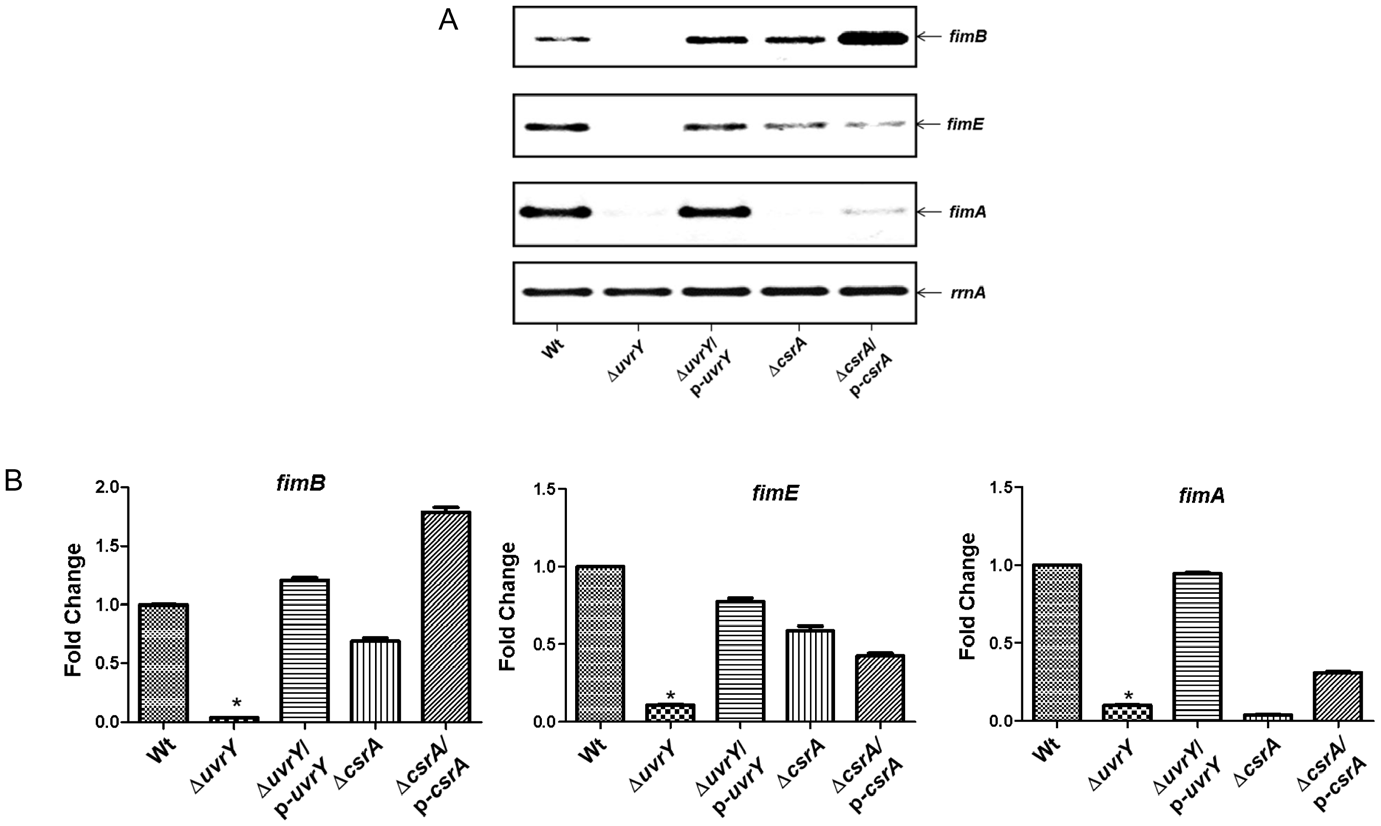
Effect of *uvrY* and *csrA* on *fimB*, *fimE* and *fimA* transcription in UPEC strain CFT073. **A**. Transcript levels of *fimB*, *fimE* and *fimA* were evaluated by quantitative RT-PCR. The *rrnA* was used as an internal control. **B**. Relative expression levels of various transcripts were calculated by normalization of the actual intensity of bands with *rrnA* control and expressed as fold change relative to the wild-type. The wild-type was set at one. Bars represent mean ± S.E.M of three replicates.

**Figure 4:**
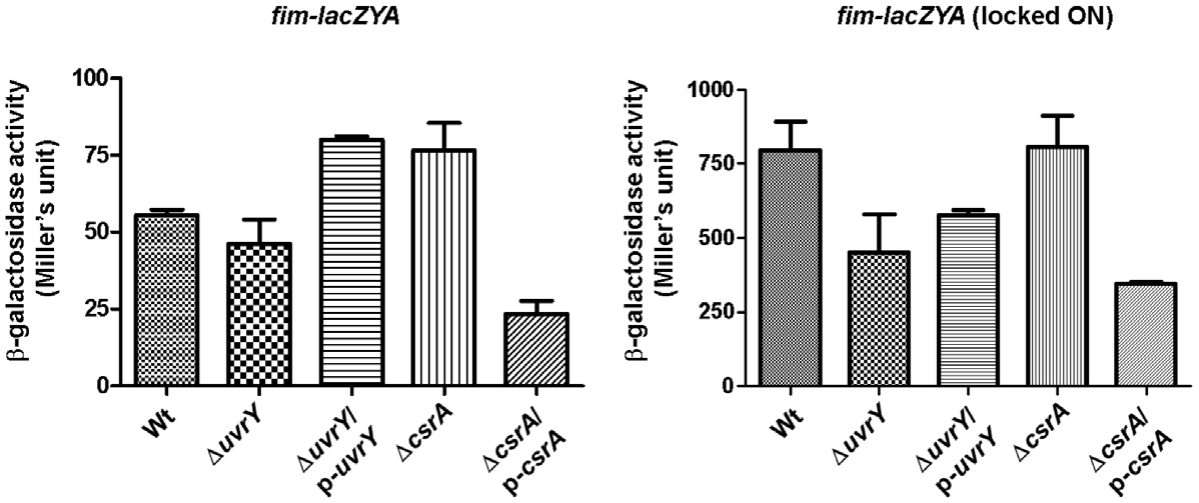
Expression of *fim* operon in K-12 strain MG1655. β-galalactosidase assay was performed in relevant genetic background harboring a single copy plasmid with *fimA-lacZYA* transcriptional fusions either at invertible or at locked ON orientation. Bars represent means ± SEM of three experiments.

Type 1 fimbriae are crucial for biofilm formation in *E. coli*. Importantly, type 1 fimbriae are associated with colonization of the bladder [5], [47]. On the other hand, Pap fimbriae (Pyelonephritis associated pilus) are associated with UPEC adherence to epithelial cells and essential for colonization in the kidneys [48]. To determine whether *uvrY* has an effect on expression of these adhesins, we evaluated the expression of *fimA* and *papA* by semi qRT-PCR in UPEC. Mutation in *uvrY* turned off the transcription of *fimA* (Fig. 3A & 3B, lane 2) and expression levels of *fimA* were restored to that of wild-type (Fig. 3A & 3B, lane 1) upon *uvrY* complementation (Fig. 3A & 3B, lane 3). In addition, the transcription of two recombinase *fimB* and *fimE* was undetectable when *uvrY* was deleted in UPEC (Fig. 3A, lane 2), however, they were restored upon complementation to the level of wild-type (Fig. 3A & B, lane 3). In UPEC, loss of *csrA* diminished the transcription of *fimB* and *fimA* and complementation of the mutant increased the expression level of both transcripts (Fig. 3B). In K-12, a plasmid based *lacZ* reporter construct fused with the entire *fim* operon was used to evaluate the effects the expression of *fim* operon on loss of *uvrY* or *csrA* gene. In K-12, *uvrY* mutation marginally reduced the expression of fimbrial operon reporter construct, while complementation of the mutant increased the expression level higher than that of the wild-type (Fig. 4, lane 2). In addition, deletion of the *csrA* increased expression of *fim-lacZYA* in reporter construct and complementation of the mutant reduced the expression (Fig. 4, lane 4 & 5). These data indicate that deletion of the *csrA* in K-12 exerts a remarkable effect on *fim* switch orientation.

### Mutation of *csrA* decreases mRNA stability of *fimA* in *E. coli* K-12

**Figure 5:**
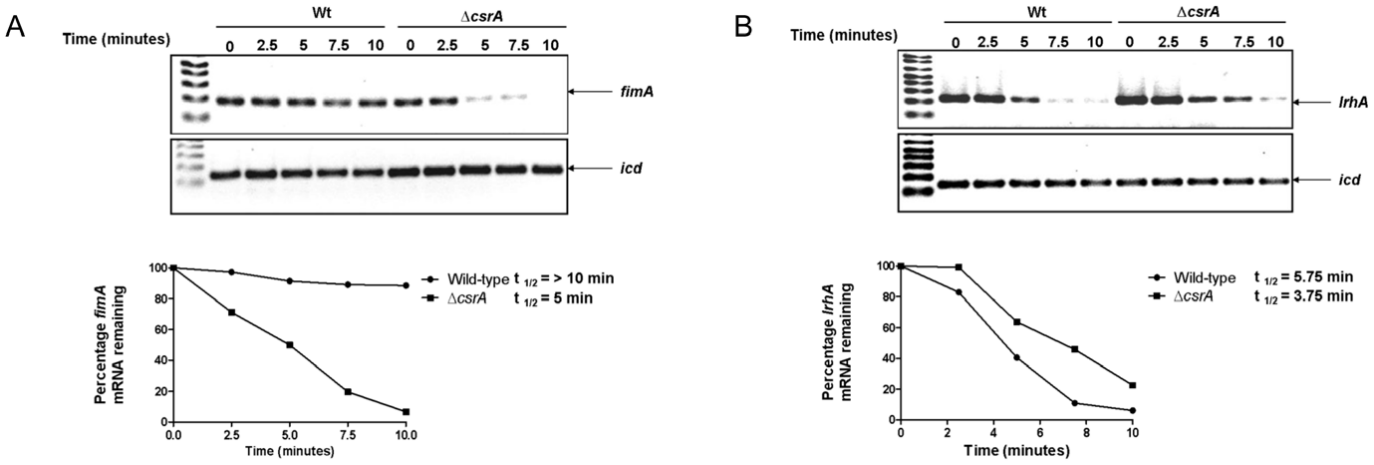
Effect of *csrA* on message stability of *fimA* (A) and *lrhA* (B) in K-12 strain MG1655. Total RNA was harvested from late log growth phase. The mRNA stability of *fimA*, *lrhA* or *icd* (housekeeping control) was assessed for 10 mins after addition of rifampicin. The relative intensities of the wild-type and the mutant was compared to the intensity of *icd* mRNA. Normalized transcript levels at time zero prior to rifampicin treatment was set at 100%. This experiment was repeated two times.

CsrA regulates various target transcripts either positively or negatively by affecting messenger RNA stability [26], [49]. We found that CsrA had a significant effect on the regulation of type 1 fimbriae in *E. coli* K-12. In contrast, this effect was not significant in CFT073 strain. To understand this effect, we further examined the role of CsrA in affecting mRNA stability of *fimA* in *E. coli* K-12. Deletion of *csrA* destabilized the *fimA* transcript in *E. coli* K-12 (Fig. 5A, lanes 6–10). This was evident by fact that the half-life of *fimA* transcript in wild-type K-12 was greater than 10 min, whereas that of *fimA* transcript in *ΔcsrA* mutant was reduced to ∼5 min. LrhA is known to repress type 1 fimbriae expression in *E. coli* [50]. Under similar conditions, the half-life of *lrhA* transcript was only mildly increased in the absence of *csrA* (Fig. 5B, lanes 6–10), but not significantly different from that of wild-type (Fig. 5B, lanes 1–5).

### Loss of *uvrY* reduces expression of several virulence specific genes in UPEC

**Figure 6:**
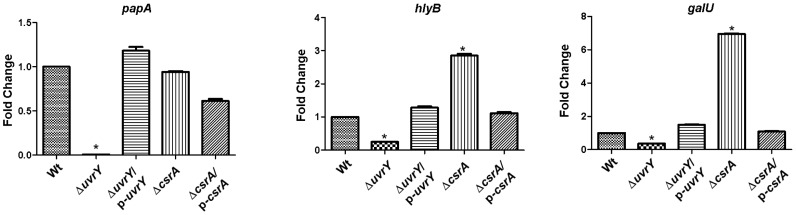
Relative expression levels of *papA*, *hlyB* and *galU* in UPEC strain CFT073. Bacterial strains were grown in LB broth for 48 hours at room temperature. Intensities of bands from agarose gels were subjected to image analysis post semi-quantitative RT-PCR from various strains. Transcript levels of *rrnA* were used as an internal control. Fold change was represented by the expression levels of genes in various strains relative to the wild-type, set at 100%. Bars represented means ± S.E.M. of three replicates.

We further evaluated the role of *uvrY* or *csrA* in expressing virulence specific genes in UPEC, such as *papA* encoding the major subunit of P fimbriae, *hlyB* involved in hemolysin export, and *galU*, an important factor for biofilm formation. Loss of *uvrY* completely abolished *papA*, reduced expression of *hlyB* and *galU* genes. The complementation experiment restored their expressions to the levels of wild-type (Fig. 6, lanes 2 & 3). However, loss of *csrA* did not increase the *papA* transcript level, whereas complementation of *csrA* reduced the *papA* transcript level relative to that of the wild-type. Interestingly, mutation in *csrA* increased expression of *hlyB* and *galU* around 3 and 7 fold respectively and upon complementation, the mutant exhibited a reduced expression of those genes similar to the level of wild-type (Fig. 6, lanes 4 and 5). These results strongly suggest the potential regulatory roles of *uvrY* and *csrA* in control of the virulence genes in UPEC.

### Loss of *uvrY* affects swarming motility in UPEC

**Figure 7:**
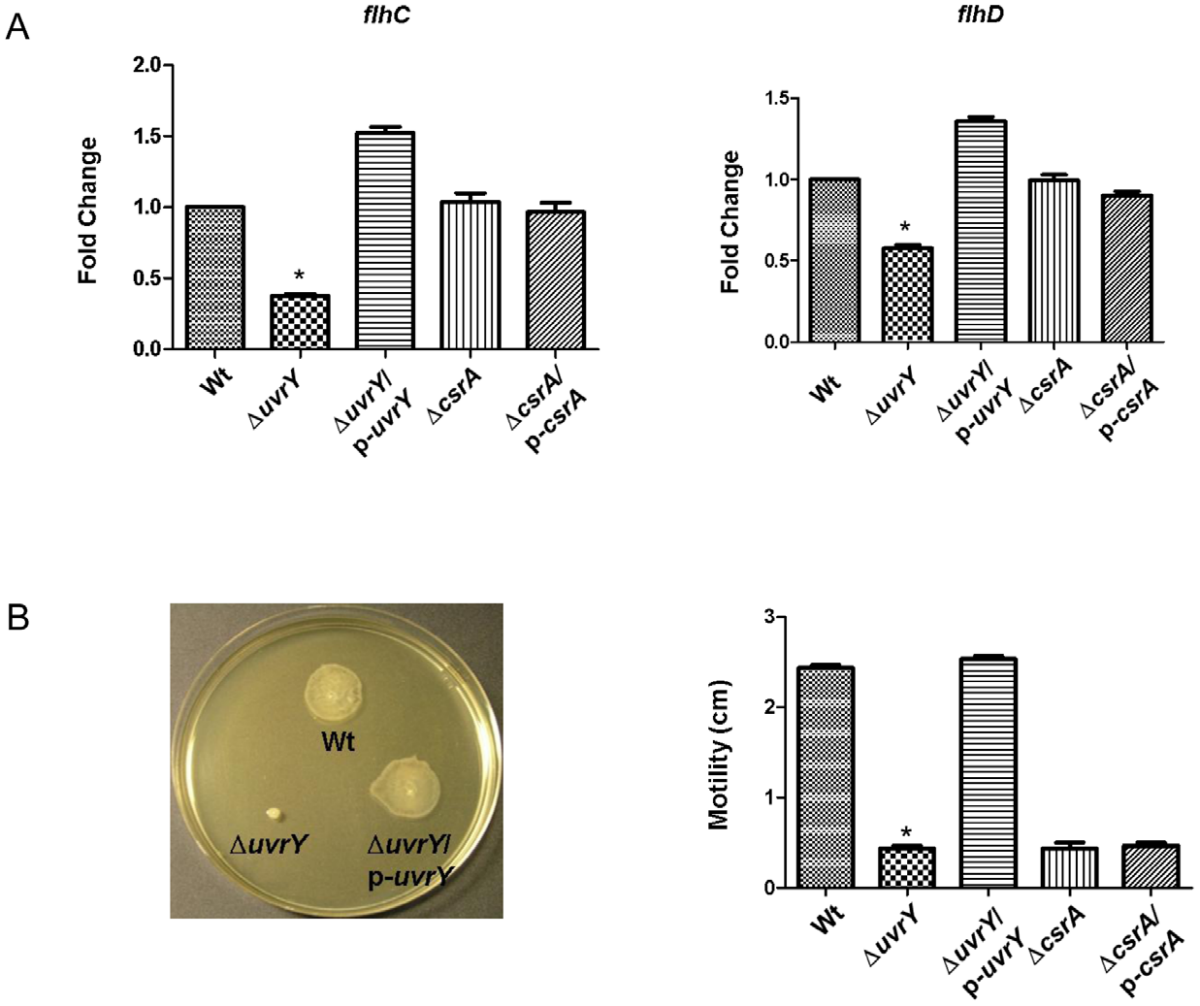
Relative expression levels of *flhC* and *flhD* transcripts (A) and swarming motility in UPEC strain CFT073 (B). Relative transcript levels of *flhC* and *flhD* was assessed by qRT-PCR with *rrnA* as an internal control and represented as fold change relative to the wild-type, set at 100%. Swarming motility was assessed on 0.6% LB agar supplemented with 0.5% glucose. The zone of swarming was measured after 16-hour incubation at 37°C. Bars indicate means ± S.E.M. of three replicates.

In *E. coli*, the flagellum is critical for initial attachment and overcoming repulsion between similarly charged bacterial and inert surfaces as well for forming biofilms [51]. Swarming motility is a flagellum-dependent form of bacterial motility that facilitates migration of bacteria on viscous substrates [52]. The swarmer cells undergo differentiation, which is characterized by elongation and an increase in flagellum number and eventual migration of the swarmer cells. In view of swarming motility in facilitating biofilm formation, we further characterized role of *uvrY* or *csrA* in regulating the expression of flagellum. In addition, the expression of flagella is dependent upon hierarchy of a cluster of genes, which are controlled by the master regulator, *flhDC*. Our results showed that loss of *uvrY* reduced expressions of *flhC* and *flhD* in UPEC (Fig. 7A, lane 2); their expression levels were restored to that of the wild-type upon the complementation (Fig. 7A, lane 3) (Fig. 7A, lane 1). Loss of *uvrY* also reduced the swarming motility in UPEC and swarming was restored to that of wild-type upon complementation (Fig. 7B). These results clearly indicate that *uvrY* influences swarming motility in UPEC.

Furthermore, the *csrA* deletion mutant in UPEC exhibited marginal effect on transcriptions of *flhC* and *flhD* genes. Over-expression of the *csrA* in the *csrA* mutant did not exert an effect on the expression levels of both *flhC* and *flhD* transcripts. We also noted that UPEC with a *csrA* deletion mutant were not as motile as the wild-type; the over-expression of the *csrA* mutant did not repair the defect in swarming motility, probably due to lack of selective pressure for plasmid maintenance in agar plates. However, the effect of CsrA could be examined by over-production in a wild-type strain as well, especially considering that p-csrA failed to complement the motility defect of the csrA mutant.

## Discussion

Biofilm formation in *E. coli* requires a set of gene expressions for facilitating its initiation, attachment and subsequent maturation. The initial attachment is mediated by several adhesins, of which, type 1 and pap fimbriae play a critical role in colonization in urinary bladder and kidneys respectively. We tested the association of the BarA-UvrY TCS in uropathogenesis of UPEC. UPEC rely on its ability to form intrabacterial biofilms in the bladder and in the pods in a polysaccharide based matrix. Such intrabacterial communities have been demonstrated to express type 1 fimbriae, antigen 43 and exopolysaccharides [53]–[56]. Our previous study has demonstrated that *uvrY* mutation in CFT073 resulted reduced colonization of the bacteria in the bladder, kidneys or in urine as compared to that of wild type [30]. In this study, we further investigated the roles of *uvrY* and *csrA* in regulating expression of genes relevant to the biofilm formation in *E. coli*.

First, we tested the association of the *uvrY* and *csrA* genes in forming biofilms of UPEC. Our findings indicated that loss of *uvrY* in UPEC significantly reduced the bacterial attachment to various abiotic surfaces. We reasoned that the lowered expression of few adhesins might be responsible for this adhesion defect based on our earlier studies with APEC & UPEC [29], [57]. We investigated the role of *uvrY* and *csrA* on type 1 fimbriae switch since type 1 fimbriae is an important factor for biofilm formation on various abiotic surfaces such as PVC, polypropylene or borosilicate glass [51]. As type 1 fimbriae is commonly present in both pathogenic and non-pathogenic strains, a comparative study in *E. coli* K-12 was also performed to further address whether such regulation holds true in both non-pathogenic and pathogenic strains. Our results indicated differential regulation between *E. coli* K-12 and CFT073 in type 1 fimbriae regulation as mediated by *uvrY* or *csrA*.

It becomes clear that *uvrY* controls transcriptions of *fimA* and *papA* which encode the major fimbrial subunit of type 1 and pap fimbriae in UPEC. Both adhesins are important for persistence of UPEC in bladder and kidneys. Furthermore, deletion of *uvrY* in UPEC also reduces expression of the master regulator, *flhC* and *flhD*. Thus mutation in *uvrY* affects the bacterial persistence in the bladder or kidneys by several possible ways. Down-regulation of both type 1 and pap fimbriae has been shown to affect adhesion and ability to persist in bladder and kidneys, respectively. The uroplakin particles of the apical cell membrane can mediate interaction with type 1 fimbriae for internalization of UPEC. Thus, mutation in *uvrY* might also compromise the uptake of UPEC into the bladder epithelial cells. Loss of *uvrY* also turns OFF the type 1 fimbriae and reduced transcription of *fimA*, suggesting that high percentage of cells in *uvrY* deletion mutant probably exists in afimbriated phase. These indicate that a successful colonization in the bladder for initiating the biofilm formation might be impaired in an *uvrY* mutant. Secondly, a mutation of *uvrY* in UPEC results in hypersensitivity to hydrogen peroxide [23]. Biofilms formed from an *uvrY* mutant might subsequently be cleared due to the phagocytosis and oxidative burst by the polymorphonuclear leukocytes. In contrast, biofilms from wild-type strain would be difficult to penetrate due to polysaccharide based matrix and protective uroplakin. Finally, flagellar motility might also play a subtle role in the fitness of UPEC, even though flagellar motility may not be absolutely critical for the virulence [58], [59]. In fact, studies have shown that the flagella is down-regulated during UPEC infection, most likely resulting from avoiding activation of toll like receptor-5 (TLR-5) mediated stimulation and release of proinflammatory cytokines such as IL-8. Flagella also promote ascendance of UPEC from the bladder and initiate upper urinary tract infections, particularly in the kidneys [60], [61]. However, transient expression of flagellar motility is thought to be important for initial colonization of UPEC in the urinary tract. Co-challenge experiments with UPEC and flagellar mutants have demonstrated that flagellar motility is important for colonization of UPEC against a flagellar mutant strain and thereby contribute to fitness of UPEC. Thus, mutation in *uvrY* might affect fitness or persistence of UPEC in bladder, kidneys, urine or formation of intracellular biofilm in the urinary tract. Mutation in *uvrY* might also compromise the attachment to such abiotic surfaces, leading to the reduced persistence of UPEC in devices such as catheters. Hence *uvrY* might be a key regulator in the pathogenesis of UPEC in the bladder or kidneys.

CsrA is a global RNA binding protein which regulates several virulence genes in *E. coli*. The CsrA protein acts on the translation of target genes and influences stability of target mRNA transcripts in positive and negative regulatory manners. Specifically, CsrA could bind at or near shine-dalgarno sequences, thus blocking ribosome loading and facilitating mRNA decay. In contrast, CsrA could also stabilize mRNA by increasing target transcript levels and subsequently increase the translation of the mRNA. Our results indicate a potential novel role of CsrA in regulation of type 1 fimbriae in *E. coli*. The regulation of type 1 fimbriae by *csrA* might be different between K-12 and UPEC. The effect of *csrA* is clearly pronounced in K-12 background as evident by impact of *fimA* transcript stability in a *csrA* deletion mutant. Earlier studies have shown that CsrA is a positive regulator of *flhDC* in *E. coli* K-12 at different growth phases under aerobic conditions [49]. Interestingly, CsrA has also been shown to negatively regulate *flhDC* in *Erwinia carotovaora* [62]. In this study, we focused on the effect of deleting *uvrY* and *csrA* on the expression of *flhDC* in UPEC CFT073 under biofilm inducing conditions. It is possible that the expression pattern of *flhDC* could be different due to differences in genetic background, growth conditions and composition of LB media, which in our experiments did not contain glucose.

In the BarA-UvrY-CsrA pathway, a deletion of *uvrY* or *csrA* would be expected to show opposite phenotypes, as UvrY stimulates the transcription of csrB and csrC non-coding RNA which sequesters free CsrA in the cell. However, in our experiments, we have observed that the deletion mutants of *uvrY* and *csrA* in UPEC are both impaired in motility. These results suggest the BarA-UvrY-CsrA pathway might be regulated differently in UPEC. However, our results are similar to a previous study on *uvrY* orthologue in Salmonella, *sirA* on motility and virulence [63]. We have also noted that mutation of *csrA* in UPEC showed growth defect and formed mucoid colonies. The mucoid phenotype could be due to the over-expression of the exopolysaccharides, such as PGA (poly-β-1,6-*N-*acetyl-D-glucosamine) and others. These phenotypes have been previously reported [64]. However, since the UvrY and CsrA are part of the same pathway, the effect of *uvrY* on biofilm formation could be affected either directly or indirectly due a downstream effect on CsrA. It is necessary to conduct experiments characterizing the phenotype of *uvrY*/*csrA* double mutant or overproduction of uvrY, csrA or both in the different mutant strains in the future.

Our results further indicate that in UPEC, CsrA represses a set of virulence genes such as hemolysin and LPS biosynthetic genes such as *galU*. This suggests that a *csrA* deletion mutant might be highly virulent in UPEC. However, CsrA may not play a crucial role in some other UPEC specific fimbrial gene regulation, such as *papA*, indicating that under certain niches such as kidneys, a *csrA* deletion mutant might not be as invasive as the wild-type.

Overall there are numerous differences between K-12 and UPEC which may explain differential regulation between these distinct strains. The genome of UPEC is relatively larger than K-12 and numerous UPEC specific genes might influence gene regulation differentially as compared to non-pathogenic strain. *E. coli* K-12 is a laboratory adapted strain which lost some important virulence genes over several passages such as O-antigen of LPS. The LPS biosynthesis requires several genes; mutations in *rfa* and *galU* genes make *E. coli* K-12 non-pathogenic. The unique virulence, such as hemolysin secretion, presence of a full-length LPS, and ability to facilitate interaction with kidney cells by p fimbriae, enables UPEC to develop biofilm. Importantly, our findings indicate that UPEC specific genes such as *papA*, *galU* and *hlyB* are also modulated by *uvrY* at the transcriptional level or by *csrA* at the post-transcriptional level. Therefore, this work highlights the importance of *uvrY* in the persistence, motility and virulence, all of which contributes to the uropathogenesis. Importantly, we can predict such regulatory role of *uvrY* in several gram-negative human pathogens, as this gene is conserved.
